# Zoonotic *Bordetella bronchiseptica* infection at the swine-human interface: unveiling the evolutionary path from an animal to a human pathogen

**DOI:** 10.1080/22221751.2026.2637286

**Published:** 2026-02-23

**Authors:** Junqi Liu, Xiaofeng Zheng, Chenghao Jia, Zhiliang Sun, Wangping Zhou, Jie Zhang, Yifeng Chen, Zijing Zhou, Yao Tian, Gang Xiao, Lifei Du, Chengming Fan, Leisheng Sun, Min Yue

**Affiliations:** a Hunan Institute of Animal and Veterinary Science, Hunan Academy of Agricultural Sciences, Changsha, People’s Republic of China; b Key Laboratory of Systems Health Science of Zhejiang Province, School of Life Science, Hangzhou Institute for Advanced Study, University of Chinese Academy of Sciences, Hangzhou, People's Republic of China; c Zhejiang University Colleague of Animal Sciences, Hangzhou, People’s Republic of China; d School of Medicine, Hunan Normal University, Changsha, People’s Republic of China; e College of Veterinary Medicine, Hunan Agricultural University, Changsha, People’s Republic of China; f The Second Xiangya Hospital, Central South University, Changsha, People’s Republic of China; g Yuelushan Laboratory, Changsha, People’s Republic of China; h School of Public Health and Emergency Management, Southern University of Science and Technology, Guangdong, People’s Republic of China

**Keywords:** *Bordetella bronchiseptica*, swine, human, zoonoses, public health, evolution

## Abstract

*Bordetella bronchiseptica*, long regarded as a veterinary pathogen, is now emerging as a zoonotic threat to humans, particularly in immunocompromised individuals. We report a sentinel event involving a synchronized *B. bronchiseptica* outbreak in swine and their human caretaker, providing a unique opportunity to examine cross-species transmission and adaptation at the genomic level. Comparative genomics revealed that the human-adapted isolate (RL57) and its swine progenitor (XX35) share an identical chromosome, with XX35 harbouring an extra conjugative plasmid. Remarkably, RL57 did not simply lose this plasmid; instead, the entire plasmid was integrated into the chromosome via site-specific recombination. This integration allowed permanent retention of plasmid-encoded virulence and fitness genes, after which the plasmid was discarded to eliminate its replicative burden – a “capture-and-discard” mechanism of evolution. Following plasmid loss, the RL57 strain exhibited hypervirulence, faster growth, enhanced thermotolerance, and increased biofilm formation, indicating successful adaptation to the human host. Plasmid loss paradoxically rewired bacterial metabolism: sulfur assimilation and sulfonate utilization pathways were upregulated to fuel host adaptation. Strikingly, despite a collapse in transcription of specific metabolic modules, translational compensation maintained high protein levels, driving robust biofilm formation and thermal tolerance. These findings reveal a previously unrecognized evolutionary strategy in which plasmid integration followed by subsequent plasmid loss amplifies pathogenicity and host adaptability. Finally, we propose a One Health surveillance triad – metagenomic tracking of plasmid–chromosome dynamics, recombination hotspot screening, and metabolic shift monitoring – to proactively identify and mitigate such zoonotic events.

## Introduction

*Bordetella* is a genus of nine closely related Gram-negative bacteria, including the human-adapted pathogens *Bordetella pertussis* and *Bordetella parapertussis* (causes of whooping cough and parapertussis, respectively) and the broad-host-range pathogen *Bordetella bronchiseptica* [1–6]. *B. bronchiseptica* is a well-known cause of respiratory disease in animals – for example, kennel cough in dogs, “snuffles” in rabbits, and atrophic rhinitis in swine [5–8]. Notably, *B. bronchiseptica* has also increasingly been reported as a zoonotic infection in humans. While human cases remain relatively uncommon, they can be severe, ranging from bronchitis to pneumonia or even fatal sepsis in immunocompromised individuals [9,10]. Understanding the evolutionary mechanisms that enable such cross-species jumps is therefore critical for assessing its public health threat [11,12].

Among the key drivers of bacterial evolution and host adaptation are plasmids, mobile extrachromosomal DNA elements that can horizontally transfer adaptive traits such as antibiotic resistance and virulence factors [13–15]. However, their carriage often imposes a fitness cost (e.g. metabolic burden), creating a persistence challenge known as the “plasmid paradox” [16–19]. How pathogens like *B. bronchiseptica* resolve this paradox to stabilize beneficial traits during zoonotic transition remains an open question.

Microbes have evolved various strategies to resolve the plasmid paradox. For example, compensatory mutations in the host genome can alleviate plasmid-induced fitness costs, and crucially, integration of plasmid DNA into the bacterial chromosome can permanently secure the plasmid’s beneficial genes while eliminating the burden of a self-replicating plasmid [18–20]. Increasing empirical evidence supports this concept, with documented cases of plasmid integration enabling pathogens to stably retain accessory genes without maintaining an extrachromosomal element [21–23]. This raises the question of whether *B. bronchiseptica* might utilize such a “capture-and-discard” mechanism during a cross-species transmission to humans.

**Figure 1.**
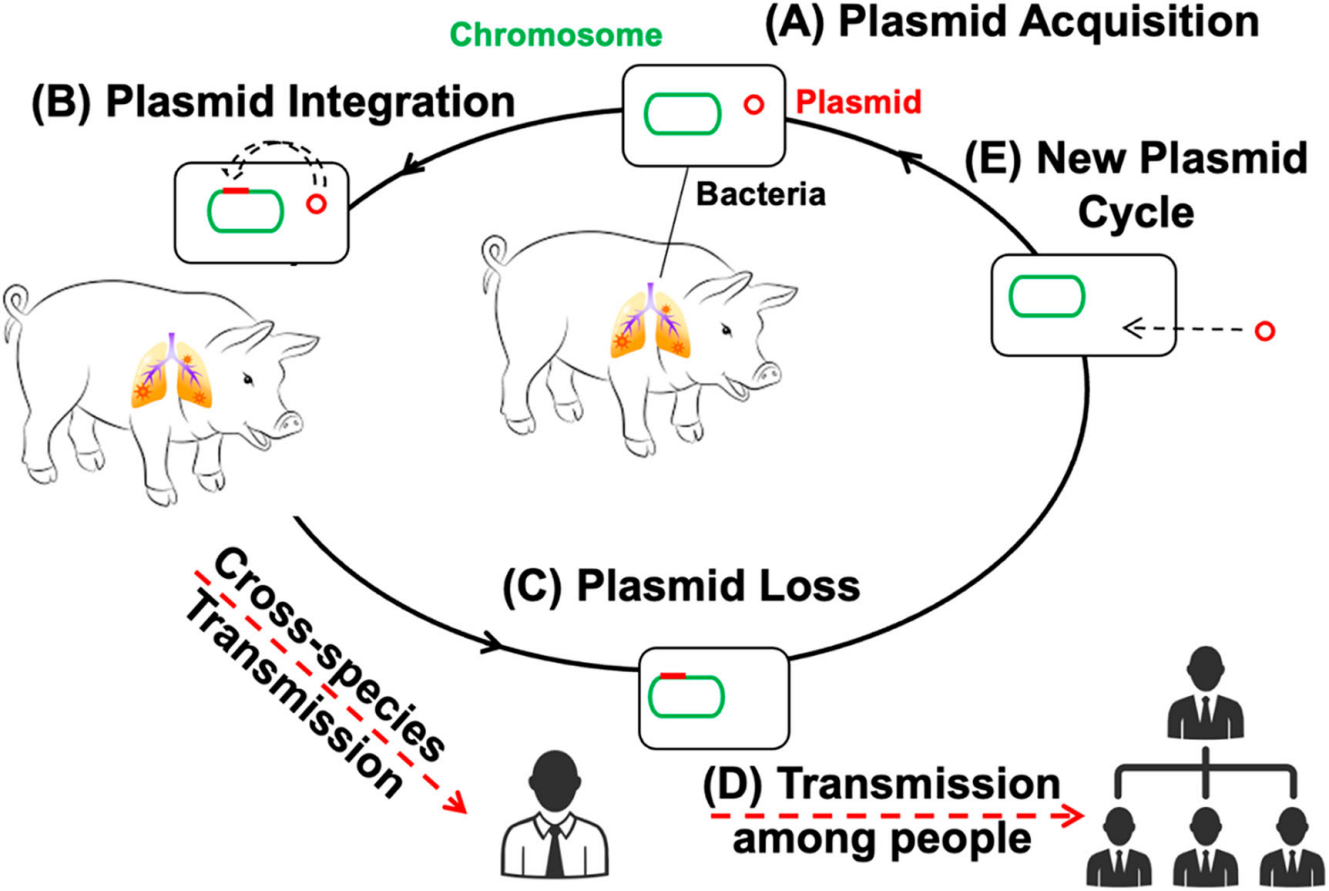
Illustration of the evolutionary pathway through which a pig pathogen transforms into a human pathogen. (**A**) Acquisition of a plasmid by bacteria under specific conditions; (**B**) Bacteria integrate the entire plasmid into the chromosome; thus, plasmid-encoded accessory traits are captured by the chromosome; (**C**) Bacteria discard the redundant plasmid to increase virulence and reduce fitness cost, facilitating human infection; (**D**) Highly pathogenic *B. bronchiseptica* with potential for human-to-human transmission, resulting in an outbreak among humans. (**E**) Bacteria might reintegrate a new plasmid and restart the cycle.

To explore this possibility, we investigated a recent zoonotic outbreak in which *B. bronchiseptica* caused respiratory disease in a swine herd and concurrently in the farm’s human caretaker. This rare sentinel event provided an opportunity to trace the pathogen’s evolutionary steps as it crossed from livestock to a human host. Comparative genomic analysis revealed that the swine isolate harboured a conjugative plasmid integrated into its chromosome, whereas the human-derived isolate had lost the extrachromosomal plasmid while retaining its genes within the chromosome. This documented “integration-then-loss” event effectively locked useful plasmid genes into the chromosome and was associated with the human-adapted strain exhibiting markedly increased virulence, faster growth, greater stress tolerance, and enhanced biofilm formation relative to its swine progenitor. These findings delineate a key evolutionary step (chromosomal capture followed by plasmid discard) by which *B. bronchiseptica* can adapt to a new host. We propose that this step could be part of a broader, cyclical “capture-and-discard” model (Figure 1), highlighting the need for One Health – oriented surveillance of such adaptive mechanisms [24].

## Methods

### Microbiological investigations

From April to September 2022, a severe respiratory outbreak struck a backyard pig farm with 798 swine in Xiangtan City, Hunan Province, China. Nasopharyngeal (n = 51) and buccal (n = 49) swabs from symptomatic pigs, plus environmental samples (drinking water n = 5, soil n = 3, fodder n = 2), were screened for key porcine respiratory pathogens using commercial kits (GeneRadar Biotechnology Corp, Xiamen, China; Wuhan Keqian Biology Co., Ltd, Wuhan, China). Targeted agents included: *influenza virus*, *porcine reproductive and respiratory syndrome virus* (PRRSV), *Actinobacillus pleuropneumoniae*, *Haemophilus parasuis*, and *Mycoplasma hyopneumoniae*. A complete list of kits used is provided (Supplementary Table S1).

### Sample collection and bacterial identification

Three symptomatic live pigs and deceased ones were transported to the Hunan Institute of Animal and Veterinary Science laboratory within 2 h, individually packaged to prevent cross-contamination. Nasopharyngeal swabs from the symptomatic farmer were sent to the Second Xiangya Hospital. Samples were streaked on tryptic soy agar (TSA) and incubated at 37°C for 24 h.

Genomic DNA was extracted using a Bacterial DNA kit (Omega Bio-tek, Norcross, GA, USA). The 16S rRNA gene and IS1001 were PCR-amplified using primers listed (Supplementary Table S2) [25,26]. Products were purified (Gel Extraction Kit, Omega Bio-tek) and sequenced (Tsingke Biotechnology Co., Ltd., Changsha, China).

### Genomic analysis

Genomes were sequenced and assembled as previously described [27]. A 400-bp paired-end library was sequenced on Illumina HiSeq 2500 (150-bp reads). PacBio data yielded one-contig genomes (plus plasmid for XX35) via Hierarchical Genome Assembly Process, refined with HiSeq data using Canu and SPAdes. Genes were predicted with Glimmer 3.02, GeneMarkS, and Prodigal; tRNA/rRNA with tRNAscan-SE v2.0 and Barrnap. Annotations used BLASTP against NR, Swiss-Prot (https://web.expasy.org/docs/swiss-prot_guideline.html), Pfam (http://pfam.xfam.org/), EggNOG (http://eggnog.embl.de/), Gene Ontology (http://www.geneontology.org/), and KEGG (http://www.genome.jp/kegg/) databases.

Comparative analyses employed MAUVE for alignment, CompareM (https://github.com/dparks1134/CompareM) for average amino acid identity (AAI), and Pyani (https://github.com/widdowquinn/pyani) for average nucleotide identity (ANI). Among 112 *B. bronchiseptica strains* (public and new), 103 passed quality control. Core SNPs were identified with Snippy (https://github.com/tseemann/snippy) (reference: GCA_030758235.1, strain XX35), recombination removed via Gubbins, and a maximum-likelihood tree built with IQ-TREE (TVM + F + R2 model, 1,000 bootstraps), visualized in iTOL. Synteny was analyzed using Mummer (Version 3.23, http://mummer.sourceforge.net/), with figures generated in BRIG and Easyfig [28,29].

### Bacterial virulence and stress assay

#### Animal virulence assessment

Seventy specific-pathogen-free female BALB/c mice (21 ± 1 g; Hunan SJA Laboratory Animal Co., Ltd., SCXK 2019-0004) were acclimatized for 7 days and randomized into seven groups (n = 10/group). Groups received intraperitoneal injections (0.1 mL) of RL57 or XX35 at 8.0 × 10⁷, 8.0 × 10⁶, and 8.0 × 10⁵ CFU in PBS, or PBS control. Clinical signs and mortality were monitored every 2 h. Procedures were approved by the Institutional Animal Care and Use Committee (HIAVS-IACUC-2022-09).

### Growth kinetics

Overnight cultures in tryptic soy broth (TSB; Oxoid, Basingstoke, UK) were diluted to an OD₆₀₀ of 0.1. Aliquots (200 μl) were dispensed into sterile 96-well plates and incubated at 37°C in an automatic microplate reader (BioTek, United States) [30]. OD₆₀₀ measurements were recorded hourly for 14 h. Growth curves were generated from triplicate biological replicates.

### Temperature stress assay

The thermal tolerance of RL57 and XX35 was assessed by incubating normalized bacterial suspensions (OD₆₀₀ = 0.1 in TSB) at 28°C, 37°C, and 42°C for 24 h. Bacterial viability was determined by plating serial dilutions onto TSA plates and counting colony-forming units (CFU). Survival rates for all test conditions were normalized to the CFU count of XX35 at 37°C. The experiment included six biological replicates per condition.

### Biofilm formation assay

Biofilm formation was assessed in 96-well plates as previously described with modifications. Overnight cultures of each strain were normalized to an OD₆₀₀ of 0.1, and 200 μL aliquots were incubated statically at 37°C for 24 h. After incubation, non-adherent cells were removed by washing with PBS. The adherent biofilms were fixed with methanol, stained with 0.1% crystal violet, and destained with 95% ethanol. The biofilm biomass was quantified by measuring the absorbance of the solubilized dye at 595 nm. Sterile TSB medium was used as a blank control. Each assay included six biological replicates.

### Transcriptomic analysis

Total RNA was extracted using Qiagen RNeasy Mini kits, and concentration and purity were assessed using a NanoDrop 2000 spectrophotometer. Strand-specific cDNA libraries were constructed following Illumina protocols and sequenced on an Illumina HiSeq platform (Shanghai Majorbio Bio-pharm Technology Co., Ltd.). Differentially expressed genes (DEGs) were identified with thresholds of |log₂FC| > 1 and adjusted *P* < 0.05.

### Proteomics analysis

Total bacterial proteins were extracted using lysis buffer (8 M urea, 1% SDS) supplemented with protease inhibitors. Protein concentration was quantified via bicinchoninic acid (BCA) assay (Thermo Scientific) and validated by SDS-PAGE. Aliquots (100 μg) were reduced with 10 mM Tris (2-carboxyethyl) phosphine (TCEP, 37°C, 1 h), alkylated with 40 mM iodoacetamide (25°C, 45 min), and digested with sequencing-grade trypsin (1:50 w/w) in 100 mM triethylammonium bicarbonate (TEAB) at 37°C for 16 h. Resulting peptides were desalted using HLB cartridges and quantified via UV absorbance (NanoDrop One, Thermo Fisher).

Samples were analyzed on a Vanquish Neo UHPLC system coupled to an Orbitrap Astral mass spectrometer (Thermo Fisher). Peptide separation employed a uPAC column (75 μm × 55 cm) with a 180-min gradient: solvent A (0.1% formic acid, 2% acetonitrile) to solvent B (0.1% formic acid, 80% acetonitrile). Data-independent acquisition (DIA) data were acquired using an Orbitrap Astral mass spectrometer operated in DIA mode. The detection was carried out over a mass range of 70–1050 m/z (MS1), and 150–2000 m/z (MS2).

Raw files were processed using Spectronaut 18 (Biognosys) with a 1% false discovery rate (FDR) threshold at both protein and peptide levels. Quantification utilized the top six peptides per protein, excluding modified/shared peptides. Differentially expressed proteins (DEPs) were identified (*P* < 0.05, fold change >1.2 or <0.8) and functionally annotated via Gene Ontology (GO) and KEGG pathway analyses using the Majorbio Cloud Platform. Protein-protein interaction networks were constructed using STRING v11.5.

### Untargeted metabolomic analysis

Log-phase bacterial cultures (OD_600_ = 0.5 ± 0.05) were harvested by centrifugation (10,000 × g, 10 min, 4°C), washed with PBS, and extracted using ice-cold methanol/acetonitrile/water (2:2:1, v/v) with 1 h sonication and −20°C incubation. After centrifugation (14,000 × g, 20 min, 4°C), supernatants were analyzed on a Thermo UHPLC-Q Exactive HF-X system with an ACQUITY HSS T3 column (100 × 2.1 mm, 1.8 μm). Mobile phases comprised 0.1% formic acid in water:acetonitrile (95:5) (Solvent A) and 0.1% formic acid in acetonitrile:isopropanol (47.5:47.5) (Solvent B). Chromatographic separation utilized a 0.4 mL/min flow rate at 40°C with 3 μL injection volume. Raw LC-MS/MS data were processed via Progenesis QI, generating a filtered matrix after removing noise, internal standards, and features with >30% RSD in QC samples. Metabolite annotation integrated HMDB, Metlin, and Majorbio databases. Multivariate analysis (PCA/OPLS-DA) and differential metabolite screening (VIP >1, *P* < 0.05) were performed using the ropls R package. KEGG pathway enrichment analysis identified biologically relevant metabolic networks through scipy.stats-based hypergeometric testing.

### Quantitative real-time PCR (qRT-PCR)

Bacterial total RNA was extracted from cultures at the mid-logarithmic growth phase (OD₆₀₀ = 0.5 ± 0.05) using the RNeasy Mini Kit (Qiagen), following the manufacturer’s protocol. The resulting RNA was reverse-transcribed into complementary DNA (cDNA) using random hexamers and a reverse transcription system. qRT-PCR was performed in triplicate with transcript-specific primers (sequences listed in Supplementary Table 3). The 16S rRNA gene was used as an internal reference for normalization. Relative gene expression levels were calculated using the 2^-ΔΔCT^ method.

### Targeted proteomic validation by Parallel Reaction Monitoring (PRM)

To validate the findings from the global proteomic analysis, a targeted Parallel Reaction Monitoring (PRM) assay was performed. Proteins were extracted from bacterial samples using a lysis buffer (8 M urea, 1% SDS) supplemented with a protease inhibitor cocktail. The extracts were clarified by centrifugation, and the protein concentration was determined using a bicinchoninic acid (BCA) assay. Protein quality was assessed by SDS-PAGE. Following reduction and alkylation, equal amounts of protein from each sample were digested with trypsin. The resulting peptides were first analyzed by data-dependent acquisition (DDA) to construct a spectral library.

For the PRM analysis, peptide samples were separated using a Vanquish Neo UHPLC system (Thermo Scientific, USA) and analyzed with an Astral mass spectrometer (Thermo Scientific, USA). The mass spectrometer was operated in positive ion mode with the following parameters: full MS scans were acquired at a resolution of 240,000 over the m/z range 350–1200. For PRM, target peptides were isolated and fragmented using higher-energy collisional dissociation (HCD). Fragment spectra were acquired at a resolution of 80,000–100,000.

The raw data were processed using Proteome Discoverer TM software (version 2.4). The quantification values for target peptides were normalized, and statistical significance was assessed using a two-sided Student’s t-test, with a *p*-value < 0.05 considered significant.

### Statistical analyses

Data are mean ± SD. Student’s t-test and ANOVA were performed in GraphPad Prism 8. Significance: *p* < 0.05.

## Results

### Clinical investigation

In 2022, a pig farm in Xiangtan, Hunan, China, experienced a significant respiratory disease outbreak, affecting 53.5% of 798 pigs. Symptoms included coughing, sneezing, dyspnea, and nasal discharge, with a total of 427 syndromic pigs and 54 deaths. Pathological examination of fatal cases revealed pronounced lung pathology, including fibrosis and collagen proliferation (Supplementary Figure 1).

Concurrently, a 39-year-old male farmer within the same farm developed respiratory symptoms, chronic cough with green sputum five days post-exposure, without fever or dyspnea. The farmer had a history of smoking and reported prior pertussis vaccination (a detail recorded given the close phylogenetic relationship between *B. pertussis* and *B. bronchiseptica*; its potential influence on this zoonotic infection remains unclear). No other personnel or family members showed symptoms, indicating a potentially direct zoonotic transmission event from swine to human, after within-farm exposure.

### Misidentification of zoonotic *B. bronchiseptica* as *B. parapertussis*

Given the swine herd’s respiratory symptoms and the farmer’s exposure history, zoonotic transmission of a respiratory pathogen was suspected. Nasopharyngeal and oral swabs from symptomatic pigs, as well as environmental samples from drinking water, soil, and feed, were collected. Initial testing with standard diagnostic kits for typical swine respiratory pathogens tested negative for common pathogens, including influenza viruses, *Actinobacillus pleuropneumoniae*, *Mycoplasma hyopneumoniae,* porcine reproductive and respiratory syndrome virus, and *Haemophilus parasuis*.

Bacterial isolates recovered from the pigs formed small, smooth, yellow colonies on solid media after 24 h at 37°C, consistent with *Bordetella spp*. Morphological and biochemical tests confirmed that the isolates were Gram-negative, oxidase-positive, and catalase-positive motile bacilli. Electron microscopy showed rod-shaped bacteria with characteristic fimbriae and flagella (Supplementary Figure 2).

16S rRNA sequencing demonstrated 100% homology with both *B. bronchiseptica* and *B. parapertussis*, while *IS1001* insertion sequence detection initially misidentified isolates as *B. parapertussis*. Further comparative genomic analysis using Average Nucleotide Identity (ANI) and Average Amino-acid Identity (AAI) confirmed a closer genetic relationship with *B. bronchiseptica* (Supplementary Figure 3). This misidentification due to shared *IS1001* sequences underscores a diagnostic gap for *B. bronchiseptica* in humans. More comprehensive methods may be needed to distinguish between these two closely related pathogens.

### Genomic investigation identified a plasmid integration event

**Figure 2.**
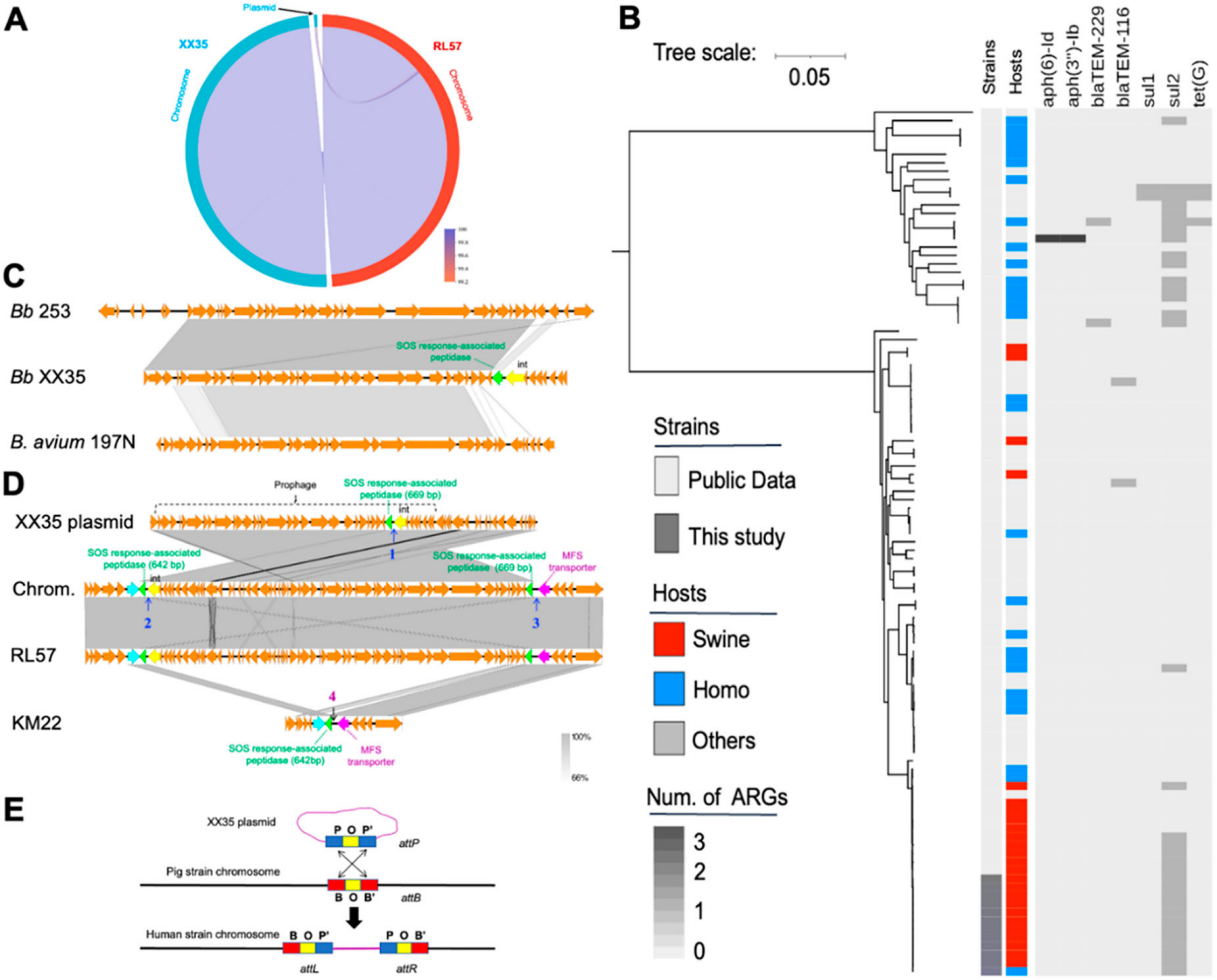
Genomic analysis of 103 *Bordetella bronchiseptica* strains. (**A**) Synteny circle between XX35 (which includes one chromosome and one circular plasmid) and RL57 (which includes one chromosome). The genome sequence of XX35, as a reference sample, was subjected to synteny analysis with RL57. XX35 and RL57 share an identical chromosomal sequence, and the plasmid sequence of XX35 is highly similar to that of the RL57 chromosome. (**B**) A phylogenetic tree was constructed for 103 *Bordetella bronchiseptica* strains using core single-nucleotide polymorphisms (coreSNPs) (Ref: GCA_030758235.1). The tree scale bar represents genetic distance, with each unit corresponding to a specific number of nucleotide substitutions per site. The heatmap on the right highlights the newly isolated strains, their host origins, and the presence of antibiotic-resistant genes in each genome. (**C**) Comparison of the prophage (CP132331:796-28633), a fragment of *B. bronchiseptica* strain 253 (HE965806:25970-59748), and *B. avium* 197N (AM167904:415990-442179). (**D**) Comparison of the XX35 plasmid (CP132331), XX35 chromosome (CP132330:1455084-1507429), RL57 chromosome (CP132332:1454971-1507181), and KM22 complete genomes (CP022962:1546432-1558192). (**E**) Site-specific recombination. One strand in the core region is cleaved at both *attB* and *attP* sites; DNA strand exchange generates a Holliday junction (HJ), and a ligation reaction between one strand from each site establishes the integrity of the DNA strand, resulting in the creation of two new hybrid sites, *attL* and *attR*.

Complete genome sequencing and resequencing confirmed that eleven swine-origin strains (XX35 – XX45) exhibited 100% nucleotide identity across both chromosomal and plasmid sequences, with multilocus sequence typing (MLST) further classifying all isolates as sequence type ST-7. The representative porcine strain XX35 (chromosome: CP132330; plasmid: CP132331) and human-derived strain RL57 (chromosome: CP132332) exhibited an identical chromosomal sequence (Figure 2(A)), suggesting a common origin. Comparative whole-genome SNP analysis indicated that RL57 displayed 17 SNPs compared to XX35 (Supplementary Table 4). A phylogenetic tree based on the core SNPs genome among our isolates and 91 *B. bronchiseptica* available in online databases revealed that all the isolates examined in this study belonged to a single clone within global genomic context and carried the sulphonamide resistance gene *sul2* (Figure 2(B)). Accordingly, porcine- and human-origin *B. bronchiseptica* from the outbreak have the exact origin. Nevertheless, XX35 carries a plasmid, whereas RL57 does not.

The circular 39.1-kb plasmid (61.6% GC content) harboured a prophage module exhibiting 99.0% nucleotide identity to *B. bronchiseptica* 253 (canine isolate; GenBank HE965806) and 91.75% similarity to *B. avium* 197N phage (avian isolate; AM167904) (Figure 2(C)). Although the eco-evolutionary dynamics of plasmid prophages remain poorly understood, there is increasing evidence that plasmid-borne prophages are stable as extra-chromosomal elements [31,32]. Strikingly, the plasmid sequence in XX35 shared 99.97% nucleotide identity and 100% coverage with the chromosome sequences in XX35 and RL57, implying that the whole plasmid sequence was integrated into the chromosome.

To further study how the plasmid was transferred to the chromosome, we first analyzed the modified XX35 chromosome sequences, in which the plasmid sequences had been deleted, using Nucleotide BLAST in the NCBI database. The plasmid-free XX35 sequence showed the highest identity with porcine strain KM22 (CP022962) (Figure 2(D)). Therefore, KM22 served as an ancestral reference for integration studies. In the circular XX35 plasmid, the gene encoding the SOS response-associated peptidase (669 bp) was adjacent to the *int* gene, encoding integrase; however, after plasmid integration into the chromosome, the 669-bp SOS-associated gene became the last gene of the XX35 plasmid genome, while *int* became the first gene (Figure 2(D)). Previous studies have indicated that the integration site is located near *int* [33]. Thus, four non-coding regions were identified as potential attachment sites by sequence alignment analysis (Supplementary Figure 4). We speculate that the arm segments of *attP* (POP') from the plasmid and *attB* (BOB') from the primitive chromosome underwent exchange, resulting in the formation of two new hybrid attachment sites, termed *attL* (BOP') and *attR* (POB'), in the XX35 chromosome (Figure 2(E)). Accordingly, the entire plasmid was integrated into the chromosome via site-specific recombination, and the chromosome captured plasmid-encoded accessory traits.

### The plasmid-into-chromosome event promotes pathogenicity

**Figure 3.**
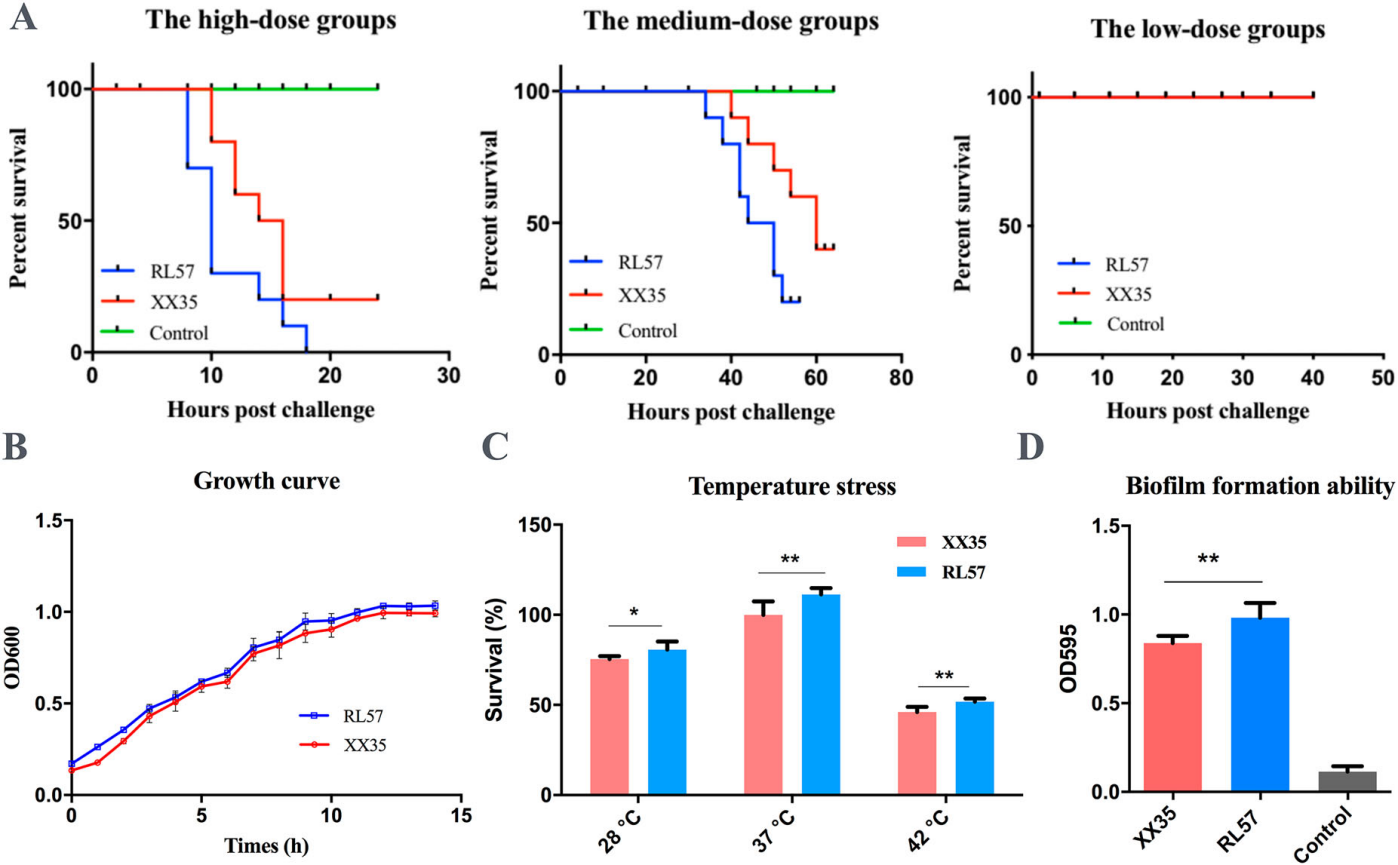
Comparison of pathobiological capabilities between XX35 and RL57. (**A**) Survival curves of BALB/c mice administered three dosages of isolate. In high-dose (8.0 × 10^7^ CFU) groups, RL57 and XX35 caused 100% and 80% mortality, respectively. In medium-dose (8.0 × 10^6^ CFU) groups, RL57 and XX35 caused 80% and 60% of mortality, respectively. In the low-dose (8.0 × 10^5^ CFU) groups, both RL57 and XX35 caused 0% mortality. (**B**) Growth curves for XX35 and RL57 cells. Statistical analysis was performed using paired *t*-tests. (**C**) Temperature stress RL57 showed higher temperature stress at 28°C, 37°C, and 42°C than XX35. Statistical analysis was performed using unpaired *t*-tests. *, *p*-value < 0.05; **, *p*-value < 0.01 (**D**) Biofilm formation ability of XX35 and RL57. RL57 showed higher biofilm-forming ability than XX35. Statistical analysis was performed using unpaired *t*-tests. **, *p*-value < 0.01.

Since the chromosome captures plasmid-encoded accessory traits via integration, the plasmid may promote bacterial fitness during infections. However, the influence of this genetic change on bacterial virulence remains unknown. To assess the virulence of individual bacterial isolates before and after zoonotic transmission, BALB/c mice were inoculated with either the plasmid-containing swine isolate or the plasmid-lacking human isolate. Results confirmed enhanced virulence in the human-origin isolate, with 100% mortality at high doses (8.0 × 10^7^ CFU) and 80% at intermediate doses (8.0 × 10^6^ CFU), compared to 80% and 60% mortality rates for swine-origin isolates at equivalent doses (Figure 3(A), Table 1). Lung pathology in infected mice included haemorrhage and thickened alveolar walls, aligning with the clinical findings stated above (Supplementary Figure 5). Furthermore, human-origin *B. bronchiseptica* also grew slightly faster, showed improved tolerance to temperature stress, and formed stronger biofilms than porcine-origin *B. bronchiseptica* (Figure 3(B–D)), indicating adaptation for human infection.

**Table 1.** Results from experimental challenges with *B. bronchiseptica* isolates in BALB/c mice.

Bacterial dose (CFU)	Strain	Number of mice	Timing of mortality post-challenge (h, number of mice)	Survival rate	Mortality
8 × 10^7^	RL57	10	8, 3; 10, 4; 14, 1; 16, 1;18, 1	0%	100%
8 × 10^7^	XX35	10	10, 2; 12, 2; 14, 1; 16, 3	20%	80%
8 × 10^6^	RL57	10	34, 1; 38, 1; 42, 2; 44,1; 50, 2; 52,1	20%	80%
8 × 10^6^	XX35	10	40,1; 44, 1; 50,1; 54,1; 60, 2	40%	60%
8 × 10^5^	RL57	10		100%	0%
8 × 10^5^	XX35	10		100%	0%
Negative controls		10		100%	0%

### Multi-omics reveals distinct patterns between human and porcine isolates

**Figure 4.**
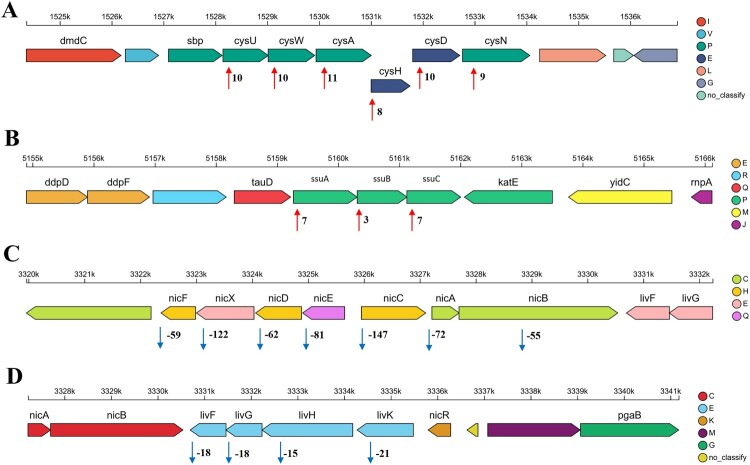
Genomic clustering of DEGs highlights co-regulated loci. (**A**) Comparative transcriptomic profiling reveals 8–11-fold upregulation of the *cysUWAHDN* in plasmid-free strain RL57 versus swine-origin strain XX35. (**B**) *ssuABC* upregulated 3–7-fold. (**C**) *nicFXDECAB* downregulated 55–147-fold. (**D**) *livFGHK* operon downregulated 15–21-fold.

To elucidate the system-level metabolic repercussions of plasmid loss, we deployed integrated multi-omics profiling, revealing unprecedented remodelling of core biochemical networks. Principal component analysis (PCA) of transcriptomic data (4,849 genes annotated) demonstrated clear segregation between RL57 and XX35 (Supplementary Figure 6A). Differential expression analysis identified 112 genes (48 upregulated, 64 downregulated; Supplementary Figure 6B), with top-enriched Gene Ontology terms implicating sulfate transport and membrane ATPase activity (Supplementary Figure 6C). KEGG pathway analysis revealed significant enrichment of differentially expressed genes (DEGs) in ABC transporters, sulfur metabolism, nicotinate and nicotinamide metabolism, and quorum-sensing pathways (Supplementary Figure 6D). Strikingly, genomic localization analysis demonstrated clustering of top-regulated DEGs: the most significantly upregulated genes mapped to contiguous genomic regions and functionally converged on sulfur metabolism (*cysUWAHDN*; 8–11 fold; Figure 4(A)) and ABC transporters (*ssuABC*; 3–7 fold; Figure 4(B)). All seven most down-regulated DEGs formed an adjacent cluster annotated to nicotinate and nicotinamide metabolism (*nicFXDECAB*; 55–147 fold downregulation; Figure 4(C)). Furthermore, the *livFGHK* operon, encoding a branched-chain amino acid (BCAA) transporter implicated in quorum sensing, exhibited robust transcriptional suppression (15–21 fold downregulation; Figure 4(D)). The Quantitative RT-PCR (qRT-PCR) results confirmed the RNA-seq data, showing consistent and significant expression changes for all tested genes (Supplementary Figure 7), thereby reinforcing the reliability of our transcriptomic profiling.

**Figure 5.**
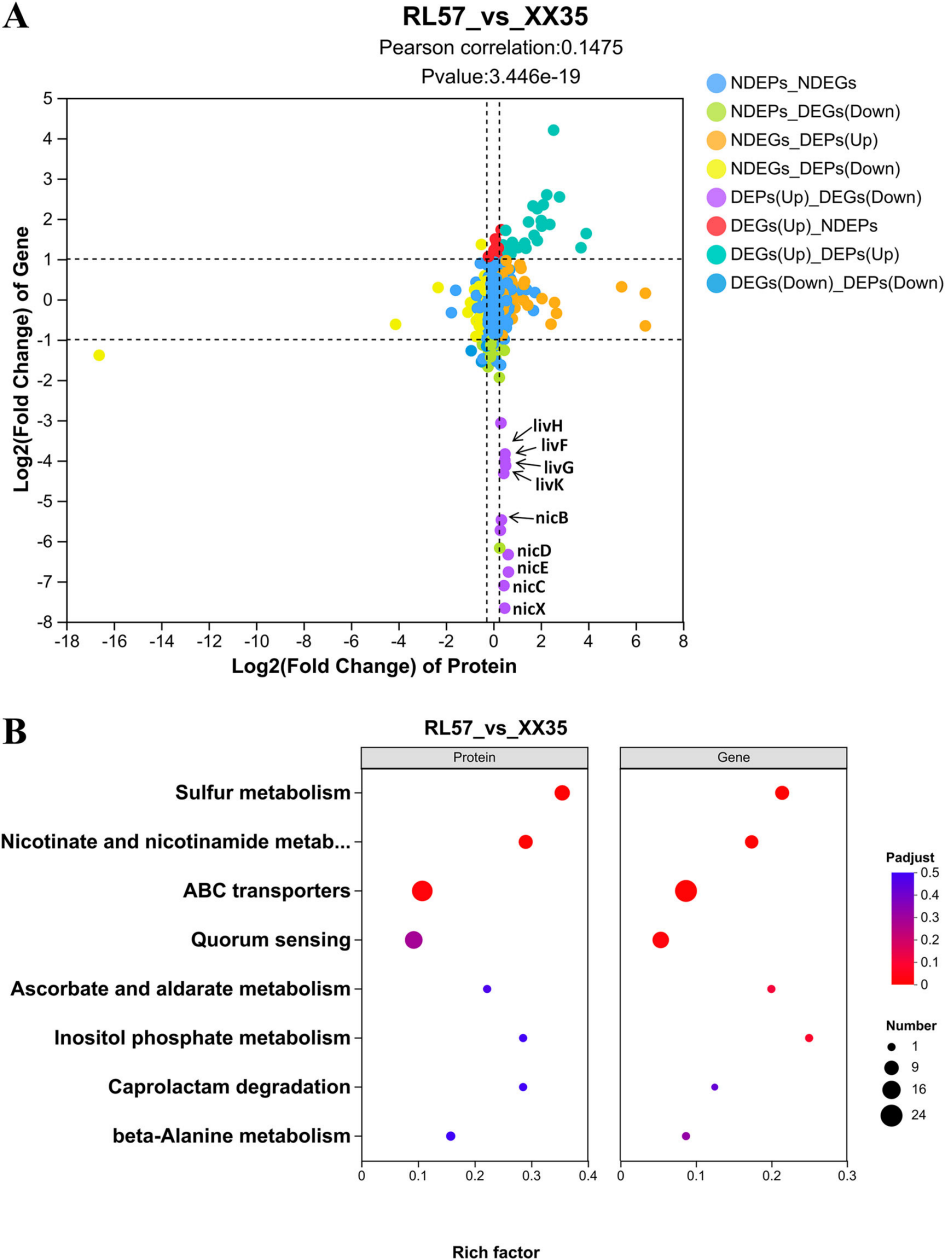
Multi-omics profiling of plasmid loss-induced adaptations in RL57 versus XX35. (**A**) Nine-quadrant plot integrating transcriptomic and proteomic profiles. The y-axis represents log₂(fold change) of gene expression; the x-axis denotes log₂(protein fold change). (**B**) Integrative pathway analysis reveals conserved alterations across transcriptome and proteome.

Proteomic profiling (3,645 proteins quantified via DIA) corroborated transcriptomic shifts, identifying 195 differentially expressed proteins (DEPs; Supplementary Figure 8A and B). KEGG enrichment analysis revealed that DEPs were predominantly enriched in sulfur metabolism, nicotinate and nicotinamide metabolism, and ABC transporters (Supplementary Figure 8C). The *cysUWAHDN* operon and *ssuABC* sulfonate utilization genes exhibited significant concordant upregulation across transcriptomic and proteomic profiling. Intriguingly, the RL57 exhibited striking RNA-protein discordance in metabolic regulation: the operon *nicFXDECAB* and *livFGHK* showed transcriptional suppression coupled with proteomic hyperactivation (Figure 5(A)). We propose that metabolic rewiring – featuring enhanced protein stabilization through putative post-translational modifications – constitutes an evolutionary adaptation mechanism enabling RL57 to optimize virulence determinants following plasmid loss. To validate the proteomic findings, we performed Parallel Reaction Monitoring (PRM) on a selection of key targets. The PRM results affirmed the expression trends observed in the proteomic data (Supplementary Figure 9, Supplementary Table 5), thereby confirming the reliability of our profiling. Comparative multi-omics analysis demonstrated substantial concordance between metabolomics and proteomics datasets in KEGG pathway enrichment profiles, with both omics layers exhibiting significant overlaps in core metabolic pathways (Figure 5(B)), underscoring RL57’s unique metabolic shifts.

LC-MS/MS-based metabolomic profiling uncovered profound metabolic remodelling in the plasmid-free RL57 strain, directly linking pathway alterations to its virulence augmentation. Multivariate analysis (PCA/PLS-DA) confirmed distinct metabolic signatures between RL57 and XX35 (Supplementary Figure 10A). Screening criteria identified 514 differentially expressed metabolites (DEMs: 458 upregulated, 56 downregulated; Supplementary Figure 10B), predominantly comprising organic acids (29.81%), lipids (27.00%), heterocyclic compounds (13.61%). VIP-ranked DEMs mapped to three virulence-critical pathways (Supplementary Figure 10C and D): Arginine biosynthesis (fuels polyamine synthesis for biofilm maturation); Glycerophospholipid metabolism (enhances membrane rigidity against host lytic factors); Purine metabolism (sustains ATP production for invasion-related energy demands). Collectively, multi-omics convergence demonstrated that plasmid loss drives systematic metabolic remodelling in *B. bronchiseptica*, enhancing environmental fitness and pathogenic potential during zoonotic transmission.

## Discussion

*B. bronchiseptica* infections, though rare in humans, pose emerging threats to immunocompromised individuals through zoonotic transmission from infected animals – particularly in settings of close human-livestock contact [34–36]. Our simultaneous isolation of *B. bronchiseptica* strains with identical core genome SNP profiles from pigs and a swine farmer provides direct genomic evidence of recent cross-species transmission event. However, establishing the exact directionality from a single time-point remains challenging. While bidirectional transmission is theoretically possible, the available epidemiological evidence strongly argues for swine as the probable origin. This conclusion is based on the marked disparity between the high asymptomatic carriage prevalence in swine populations (18.6%) [37] and the extreme rarity of human clinical cases [10,38], coupled with the absence of any documented instances of human-to-swine transmission. Therefore, although not definitively proven, the confluence of evidence strongly favours a porcine-to-human spillover in this instance, confirming *B. bronchiseptica*’s zoonotic potential at the animal-human interface.

This study uncovers a plasmid-chromosome dynamic driving *B. bronchiseptica* zoonotic adaptation. Our genomic and phenotypic data directly document a complete “integration-then-loss” cycle in this transmission event: (1) the progenitor swine strain carried a chromosomally integrated plasmid, (2) site-specific recombination led to the loss of the extrachromosomal plasmid in the human-adapted strain while preserving its genes on the chromosome, and (3) this genomic reorganization conferred a suite of adaptive phenotypic advantages (Figure 1). We therefore posit that the observed mechanism represents one iteration of a potentially cyclical “acquire-integrate-discard” dynamic. Such a strategy would enable bacterial pathogens to trial and permanently acquire adaptive traits from mobile genetic elements. Crucially, the plasmid streamlining we observed represents a critical zoonotic adaptation process where chromosomal retention of beneficial genes synergizes with metabolic burden reduction to drive cross-species transmission success.

BLAST analysis indicated that plasmid-borne prophages in our isolates showed high sequence homology with those strains isolated from dogs and turkeys (Figure 3(C)), suggesting that the plasmid-chromosome integration event may facilitate cross-species infections. This means that bacteria could acquire some novel biological properties in response to complex environments, particularly during cross-species infections.

Given that plasmid loss is conducive to bacterial evolution, why are plasmids widely spread across bacteria? Although the specific mechanisms are poorly understood, the paired concepts of “plasmid persistence” and the “plasmid paradox” might provide a framework to explain this contradiction. (i) “Plasmid persistence” overcomes the “plasmid paradox”: Plasmids carrying beneficial genes can be horizontally transferred across diverse bacteria [16,17]. To endure within hosts, bacteria employ strategies to mitigate the paradox, such as compensatory evolution and infectious transmission [17,18], allowing plasmids to persist long-term. (ii) The “plasmid paradox” drives integration as an evolutionary solution: Despite their benefits, plasmids impose a fitness cost [39]. To capture plasmid-borne traits while alleviating this burden, a widely observed strategy is the integration of plasmid genes into the chromosome， a phenomenon documented in various Gram-negative pathogens (e.g. *Salmonella*, *Yersinia*, and members of *Enterobacteriaceae* harbouring IncF or IncI plasmids) to stabilize resistance or virulence determinants [40,41]. Our study extends this concept by documenting a complete “capture-integrate-discard” cycle in *B. bronchiseptica*. We propose that after integration, the precise discarding of the redundant plasmid (and potential acquisition of new ones) initiates an iterative evolutionary cycle. Bacteria proficient in such cycles could exhibit heightened genomic plasticity and diversification, thereby expanding their adaptive potential. The *B. bronchiseptica* case exemplifies how this general strategy can be specifically co-opted for host adaptation during zoonotic transmission.

Critically, we discovered that plasmid loss triggers profound alterations in bacterial biological properties. The plasmid-free strain RL57 exhibited markedly enhanced phenotypes, including accelerated growth kinetics, expanded thermal tolerance spectrum, reinforced biofilm formation capacity, and increased lethality in murine models. Contrary to the classical “plasmid-encoded virulence” paradigm, we elucidate a chromosomal optimization mechanism through site-specific recombination capturing plasmid-derived fitness determinants. This evolutionary strategy achieves functional internalization with burden expulsion, conferring a competitive advantage in host-mimicking conditions. Our findings redefine plasmids as transient evolutionary catalysts rather than static virulence repositories in bacterial host adaptation.

The evolutionary success of pathogenic bacteria hinges on metabolic plasticity for host adaptation [42]. Our integrated multi-omics analyses indicate that plasmid loss drove metabolic reprogramming in RL57, central to which was a coordinated hyperactivation of sulfur assimilation – a pathway fundamental to virulence via synthesis of cysteine, iron-sulfur clusters, and antioxidants [43–45]. This involved significant upregulation of the sulfate assimilation operon *cysUWAHDN* (8–11-fold) and the sulfonate transporter *ssuABC* (3–7-fold), enhancing the strain’s ability to acquire and utilize sulfur sources under host restriction [7,46]. This rewiring likely augments production of critical compounds like glutathione, directly contributing to RL57’s observed accelerated growth, robust biofilm formation, and elevated murine lethality. Thus, hyperactivated sulfur metabolism constitutes an adaptive trait refined through plasmid integration and subsequent loss.

Furthermore, a transcriptome-proteome discordance in *nicFXDECAB* (nicotinate salvage) and *livFGHK* (quorum-sensing transporter) genes suggests post-transcriptional regulation. The resulting proteomic hyperactivation may fuel NAD^+^ – dependent energy generation [47] and enhance biofilm signaling, representing a resource-reallocation strategy that optimizes virulence determinants after plasmid loss.

The observed transcriptome-proteome discordance in *nicFXDECAB* (nicotinate salvage) and *livFGHK* (quorum-sensing transporter), featuring transcriptional suppression yet proteomic hyperactivation, implies post-translational regulatory mechanisms such as enhanced protein stability or translational efficiency. This paradoxical regulation may drive virulence evolution in plasmid-free RL57: (i) NAD^+^ metabolic rewiring. Elevated NAD^+^ pools from *nicFXDECAB*-mediated hyperactivation fuel proton motive force generation through the cytoplasmic membrane electron transport chain [47], thereby driving ATP synthase activity. (ii) Biofilm-centric virulence. Elevated protein levels of the ABC transporter *livFGHK* may enhance the transport of quorum-sensing signaling molecules, promoting biofilm formation – a virulence-associated trait linked to immune evasion. (iii) Evolutionary trade-off. Resource reallocation through transcriptional frugality, sacrificing gene expression while preserving protein function, optimizes virulence determinants post-plasmid loss, representing a metabolic adaptation strategy.

The diagnostic misidentification of *B. bronchiseptica* as *B. parapertussis* using *IS1001*-based assays [48–50] underscores critical limitations in current clinical detection paradigms. Given that *IS1001* is a standard PCR target in many routine clinical laboratories for diagnosing parapertussis, this creates a systematic and likely underappreciated risk of misdiagnosing zoonotic *B. bronchiseptica* infections in humans. This diagnostic gap carries significant therapeutic implications: misclassified patients prescribed macrolides face treatment failure due to *B. bronchiseptica*’s intrinsic resistance, potentially exacerbating antimicrobial resistance. We advocate for multiplex diagnostic frameworks incorporating species-specific markers, alongside metagenomic sequencing to resolve ambiguous cases and detect emerging recombinant strains.

This study demonstrates natural zoonotic transmission of *B. bronchiseptica* from swine to humans, underscoring the spillover risk at intensifying human-animal interfaces. It validates the critical role of the One Health framework, which recognizes the interconnectedness of human, animal, and environmental health [51], in addressing such zoonotic respiratory pathogens [53–55]. Our findings reveal that IS1001-based PCR can misdiagnose animal-associated pertussis-like illness as *B. parapertussis*, missing zoonotic *B. bronchiseptica*. The identified plasmid integration-loss cycle – a dynamic mechanism for host adaptation and virulence gain (e.g. in the hypervirulent RL57 strain) – highlights the need to monitor genomic recombination hotspots. To preempt such threats, proactive One Health strategies integrating genomic surveillance (e.g. phylodynamic tracking) and interdisciplinary collaboration are essential (Supplementary Table 6) [51,52].

## Author contributions

**Junqi Liu**: Conceptualization, Methodology, Formal analysis, Writing – original draft, Writing – review & editing. **Xiaofeng Zheng**: Conceptualization, Methodology, Supervision. **Chenghao Jia**: Methodology, Supervision. **Zhiliang Sun**: Methodology, Supervision. **Wangping Zhou**: Formal analysis. **Jie Zhang**: Formal analysis. **Yifeng Chen**: Formal analysis. **Zijing Zhou**: Investigation. **Yao Tian**: Formal analysis. **Gang Xiao**: Investigation. **Lifei Du**: Investigation. **Chengming Fan**: Conceptualization, Funding acquisition, Project administration. **Leisheng Sun**: Conceptualization, Funding acquisition, Project administration. **Min Yue**: Conceptualization, Methodology, Funding acquisition, Formal analysis, Writing – original draft, Writing – review & editing, Project administration.

## Supplementary Material

SupplementaryDocuments.pdf

## Data Availability

The genome sequences were submitted to GenBank and assigned the following accession numbers: CP132330 (XX35, chromosome), CP132331 (XX35, plasmid), and CP132332 (RL57, chromosome). The nucleotide sequences of 16S rRNA were submitted to GenBank under accession number OR553882. Original whole-genome resequencing data of XX36, XX37, XX38, XX39, XX40, XX41, XX42, XX43, XX44, and XX45 were deposited in the Sequence Data Archive under accession number PRJNA1100293. The transcriptome sequenceing (RNA-Seq) raw data were deposited in the NCBI Sequence Read Archive (SRA) with the accession numbers SAMN41704787, SAMN41704788, SAMN41704789, SAMN41704790, SAMN41704791, and SAMN41704792. The mass spectrometry proteomics data have been deposited to the iProX Consortium (https://www.iprox.cn) with the dataset identifier IPX0014095000. The metabolomics data have been deposited to MetaboLights repository (https://www.ebi.ac.uk/metabolights) with the study identifier MTBLS13448.
