# Supplementary material for: Zoonotic Bordetella bronchiseptica infection at the swine-human interface: unveiling the evolutionary path from an animal to a human pathogen
Source: Emerg Microbes Infect. 2026 Feb 23;15(1):2637286. doi: 10.1080/22221751.2026.2637286 (PMC12973838; doi:10.1080/22221751.2026.2637286)

Supplementary Table 1. Kits used for pathogen detection

Diagnostic kit	Targeted pathogen	Manufacturer
Swine Influenza Reagent Set	*Influenza virus*	GeneRadar Biotechnology Corp
PRRSV-CN Detection Kit	*Porcine reproductive and respiratory syndrome virus*	GeneRadar Biotechnology Corp
Actinobacillus pleuropneumoniae ApxIV-ELISA antibody test kit	*Actinobacillus pleuropneumoniae*	Wuhan Keqian Biology Co., Ltd
Real-Time PCR *Haemophilus parasuis* Reagent Set	*Haemophilus parasuis*	GeneRadar Biotechnology Corp
M. hyopneumoniae Reagent Set	*Mycoplasma hyopneumoniae*	GeneRadar Biotechnology Corp

Supplementary Table 2. Primers used for PCR amplification

Gene	Primer sequence (5’-3’)	Product size
16S rRNA	(F): AGYGGCGRACGGGTGAGTAA	1400 bp
	(R): CCATTGTAGCACGTGTGTAGC	
IS1001	(F): CCGCCTACGAGTTGGAGA	493 bp
	(R): CCGCTTGATGACCTTGATAG	

F: forward primer; R: reverse primer

Supplementary Table 3. Primer sequences for qRT-PCR

Gene	Primer F (5’-3’)	Primer R (5’-3’)
16s RNA	TACCGGGCTAGAGTGTGTCA	CATCGGTGTTCCTCCGCATA
cysU	CTTCTTTCCAGGGAAGCGGT	GGCTTCTGGGATACGGTCAC
cysW	GTTGCAGGACCATGACTGGA	ATGAACGGGAACGTGACGAA
cysA	CCAGGAAGCCGTACACGAAT	ACCGCGTTGTCCTGATGAAC
cysH	GATCATCGCGCGGCAGTAT	ATCCAGGGTGAACACCTCCA
cysD	CCGATTTCCAACTGGACCGA	CGGGCATGGCTGTAGTAGAT
cysN	GACTTCTCGTTGCTGACCGA	GTCGGCGATGATGAACTTGC
ssuA	ATCCGCATCGCCGAACAATA	TGCCTGCCGTGCTTTTCTAT
ssuB	GCCACGCACTATCTGGAGAA	GGCTCGTCCATCAGCAGAAT
ssuC	GCTGACGGCGATGTTCAATC	ATTCACAGCCATTGCCCACA
nicF	GAACCCGTTGACGAAGTCCA	AATGATCTGGGCAGCTACGA
nicX	AGTCCAGTACCTTGCCCTCG	TGCCATATCGACATTCCGCT
nicD	TCCTCGTGAAACCCCTCGAA	CGAGCAGATGAAGGCGTACT
nicE	TAGCCCTCGTTCCCGATGTA	GGTCGATGGGTTGAAGGTGA
nicC	GGCCCCAACGTGATGAAGAT	TACCAGTAGTCCGGATGCGA
nicA	TACTGCCTCAACGGCATGAT	TTTCGATATGGGTGCCGCAG
nicB	CCTGATCCAGGTCATCCTGC	TATTGCACCAGTTCGGCGAT
livF	AAATGCACGTTCTGCTCCGA	TGATGGGCAACCCTTTCCTG
livG	CACTTCCTGCACCTTGGGAT	CTGTTCACCGAGCACAGCAT
livH	ACCAGGTTGTAGACCGGGTA	CTGGTGCTGGTCATCAACGA
livK	AAATCGACCATCACGCCCTT	CTGAAGCTGACCATCCCGTT

Supplementary Table 4. A Summary of SNPs in RL57 compared to XX35

CHROM	POS	TYPE	REF	ALT	FTYPE	EFFECT	GENE	PRODUCT
CP132330.1	1021237	snp	C	A				
CP132330.1	1359536	ins	C	CG				
CP132330.1	1416543	ins	A	AG				
CP132330.1	1486311	del	TA	T	CDS	frameshift_variant c.985delA p.Arg329fs		Phage capsid protein
CP132330.1	1492100	ins	C	CG				
CP132330.1	1553532	ins	C	CGG				
CP132330.1	1807198	del	CCCCCCCCCCCCCCCCC CCCCCCCCCCCCT	C	CDS	frameshift_variant c.273_301delCCCCCCCCCCCC CCCCCCCCCCCCCCCCT p.Pro92fs	bapC	autotransporter BapC
CP132330.1	1988758	del	TGG	T				
CP132330.1	2360017	del	GC	G				
CP132330.1	2914721	del	GC	G				
CP132330.1	3120974	ins	G	GC				
CP132330.1	3258063	ins	C	CG				
CP132330.1	3621250	ins	C	CCAA				
CP132330.1	3621322	ins	T	TC				
CP132330.1	3936699	ins	C	CG				
CP132330.1	3997961	ins	C	CCGG	CDS	conservative_inframe_insertion c.168_169insCCG p.Gln56_Gly57insPro		Hydrolase
CP132330.1	4620382	ins		TC				

Supplementary Table 5. PRM-based relative quantification of target proteins

Protein	Peptide	PRM_FC (RL57/XX35)	Proteomics_FC (RL57/XX35)	Trend
CycW	LGEVALPAPDLPEADNQR	3.93919526158851	4.31904984872331	consistency
CycN	GVFADQLDAISR	5.92349056276624	6.93699575440177	consistency
NicX	IEGGLDAELLR	1.44913572192928	1.39905143862882	consistency
NicD	FGEQFDTYILDVR	1.44376761801233	1.54784441942553	consistency
NicE	ALEIADNLEVGR	1.67100286722899	1.56039901570663	consistency
NicC	DPLPLWSR	1.47743240640699	1.36827307342235	consistency
NicB	GFAYASVIDDSVDPPLR	1.24141113322233	1.27156606432421	consistency
LivF	DIAGLQPFEIAR	1.32099085199309	1.40641517028876	consistency
LivG	VQEVYFGTGK	1.29857985965985	1.43986470257027	consistency
LivH	QAGQWWSAR	1.4956135241822	1.41335031454998	consistency
LivK	LTIPFGPIEYR	1.38025432101031	1.36098429821102	consistency

Supplementary Table 6. A proposed One Health surveillance framework for preempting *B. bronchiseptica* zoonotic adaptation

**Surveillance Component**	**Target / Focus**	**Proposed Methods / Tools**	**Primary Objective**
1. Genomic Surveillance of Plasmid-Chromosome Dynamics	Plasmid integration, excision, and loss events; mobilization of accessory genes.	Longitudinal whole-genome sequencing of clinical & veterinary isolates; long-read sequencing to resolve structures.	To identify and track high-risk clones that are stabilizing adaptive traits via genomic reorganization.
2. Recombination Hotspot Diagnostics	Genomic islands, attachment (att) sites, and mobile genetic elements facilitating gene flow.	In silico prediction of recombination sites; PCR-based screening of key genomic loci.	To enable proactive monitoring of genomic regions with high potential for pathogenicity island acquisition or loss.
3. Global Metabolic Shift Monitoring	Metabolic rewiring (e.g., sulfur assimilation, ABC transporters) linked to host adaptation.	Transcriptomic (RNA-seq) and proteomic profiling of isolates from different hosts.	To detect phenotypic signatures of host adaptation that may precede or accompany virulence augmentation.

Supplementary Figure 1. Hematoxylin and eosin staining.

(**A**) Lung tissue shows serosal thickening and connective tissue hyperplasia (black arrow). (**B**) Alveolar epithelial cells are irregular and partly necrotic (green arrow). The alveolar cavity narrowed or disappeared, accompanied by extensive inflammatory cell infiltration (black arrow). (**C**) Lung tissue shows large areas of alveolar fibrosis and a large amount of collagen fibre hyperplasia (black arrow); alveolar epithelial cells are disorganized (green arrow). (**D**) Hepatocyte necrosis and nucleolysis (black arrow). The hepatic sinuses show congestion and dilatation (green arrow).

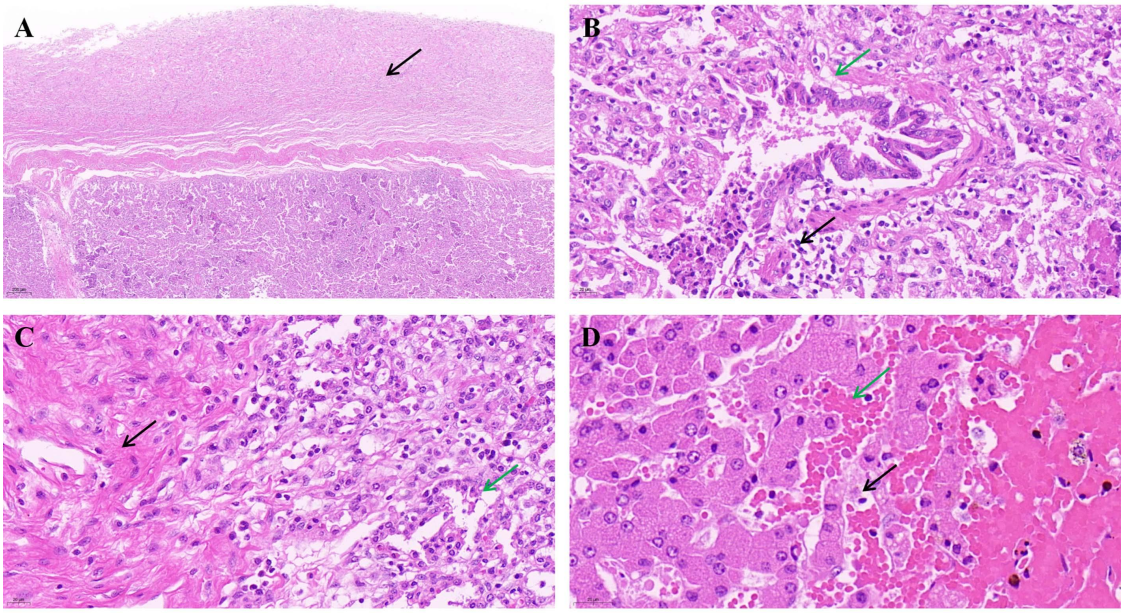

Supplementary Figure 2. Morphological characteristics of isolates.

(**A**) Colonies on tryptic soy agar after 24-h incubation at 37 °C. (**B**) Isolates are Gram-negative bacteria as determined by Gram staining. (**C**) The scanning electron micrograph and transmission electron micrograph of the human-origin strain RL57. (**D**) Scanning electron micrograph and transmission electron micrograph of porcine-origin strain XX35. These are rod-shaped bacteria with fimbriae and flagella.

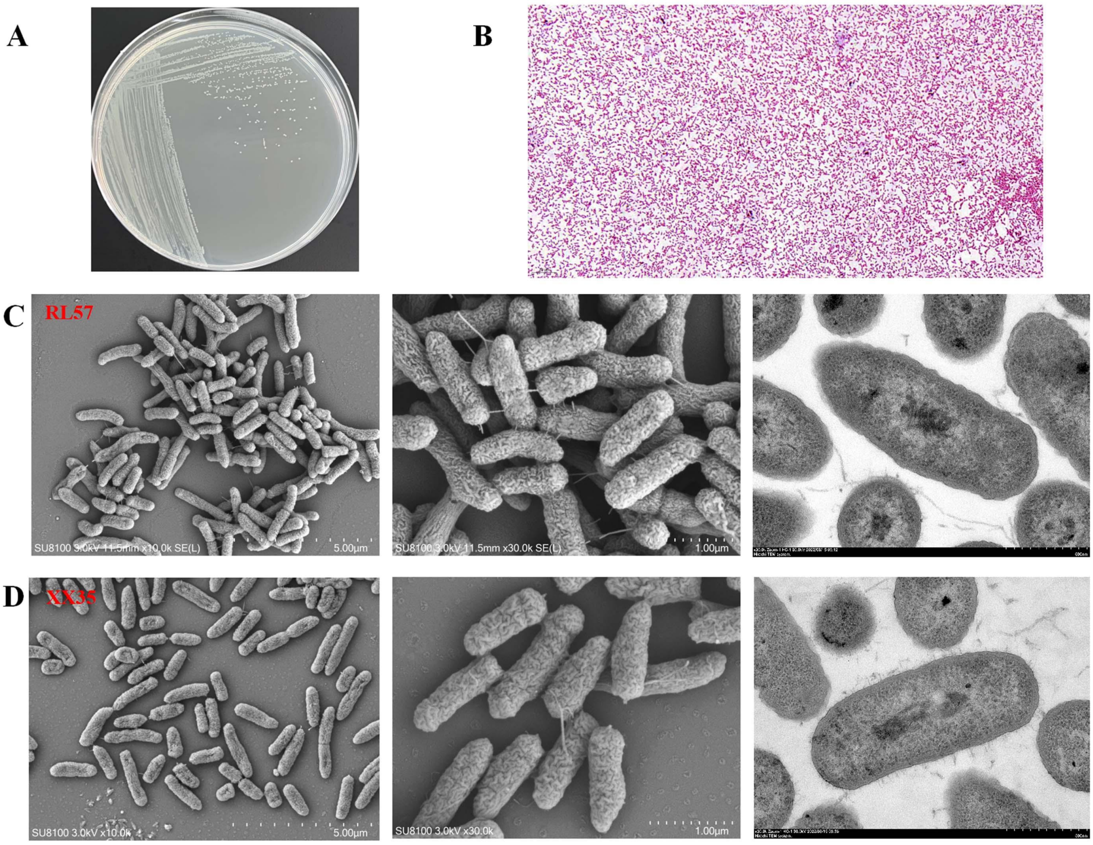

Supplementary Figure 3. ANI and AAI analysis.

(**A**) Isolates are closest to *B. bronchiseptica* strains D755 and KM22 based on Average Nucleotide Identity analysis using the Pyani software (https://github.com/widdowquinn/pyani). (**B**) Isolates are most similar to *B. bronchiseptica* strains D755 and KM22 based on Average Amino-acid Identity (AAI) using the CompareM software (https://github.com/dparks1134/CompareM).

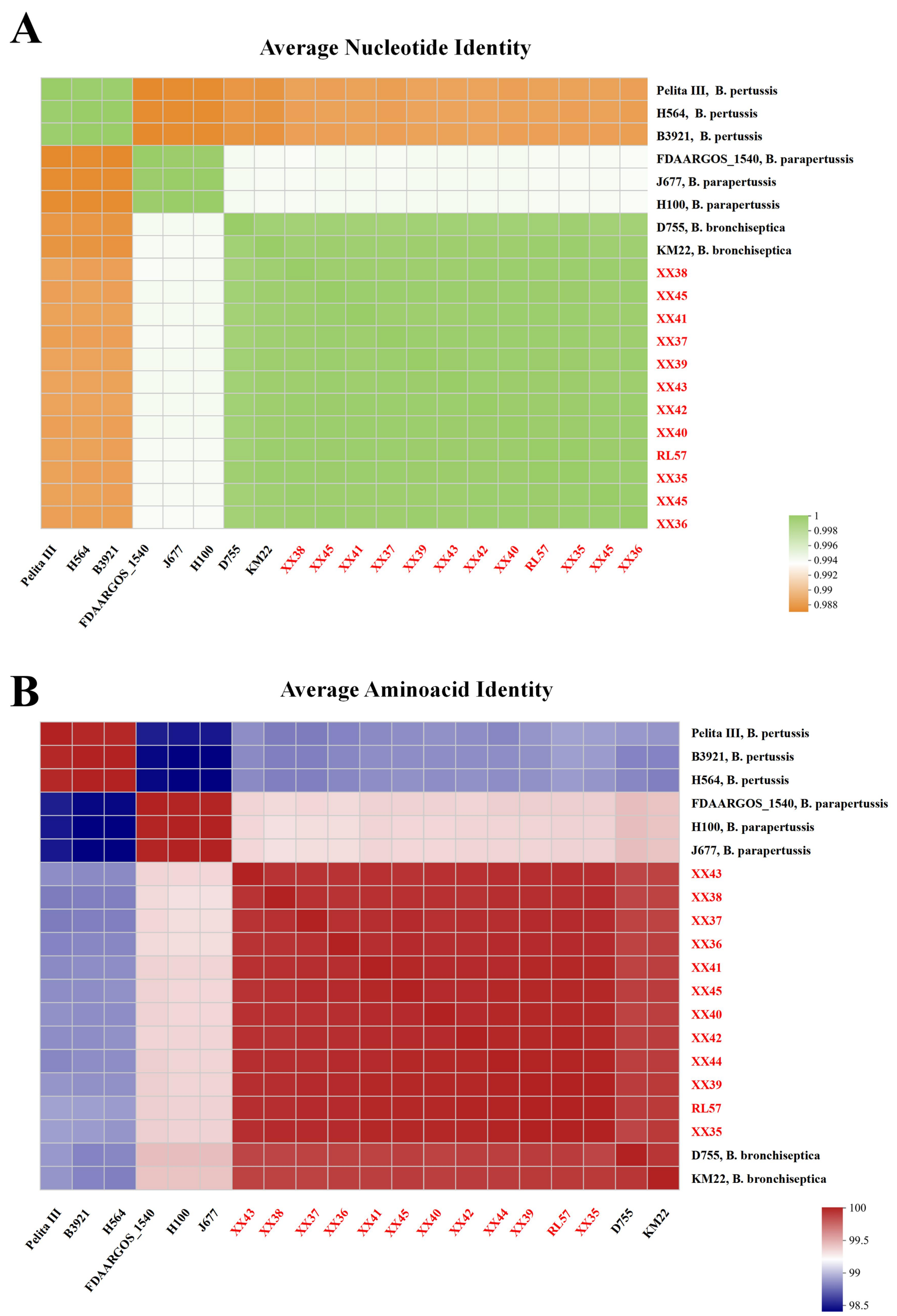

Supplementary Figure 4. Nucleotide sequences of the attachment sites

The DNA attachment sites are referred to as *attP* (POP’) and *attB* (BOB’) in XX35 plasmid and the primitive XX35 chromosome, respectively. The integrated plasmid is bounded by *attL* (BOP’) and *attR* (POB’) in the XX35 chromosome. The core sequences, which are the sites of strand exchange, are indicated in yellow. Through sequence alignment analysis, several key observations were made: (1) the putative core sequences (O), which are the same in all four attachment sites, were identified; (2) the P and B arms demonstrated sequence homology, while P’ and B’ arms exhibited sequence divergence; (3) on the left of core sequences, *attP* and *attR* shared the P arm (P), while *attB* and *attL* shared the B arm (B); (4) on the right of core sequences, *attP* and *attL* shared the P’ arm (P’), while *attB* and *attR* shared the B’ arm (B’).

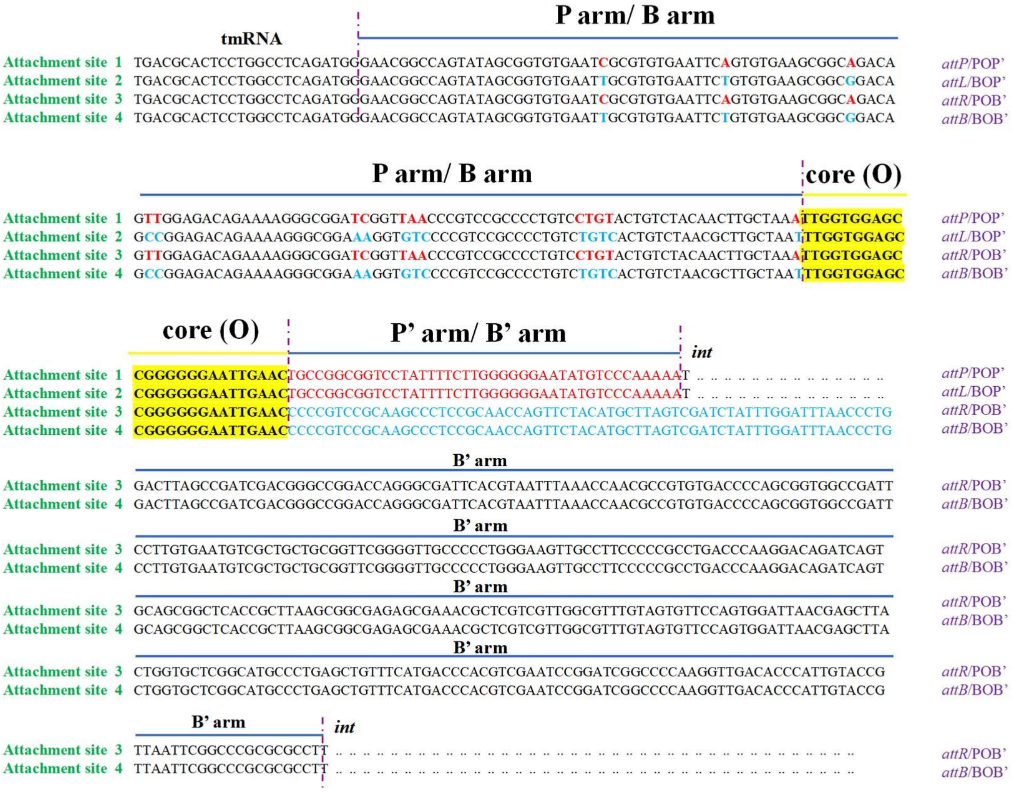

Supplementary Figure 5. Lesion gross and pathological observation in the lungs. The lungs exhibit haemorrhage (green arrow) in the high-dose and medium-dose groups. Pathological staining with hematoxylin and eosin staining showed that the alveolar walls are thickened and the capillaries in the alveolar walls are congested with many red blood cells (yellow arrow). No significant lesions were observed in the low-dose groups and control groups.

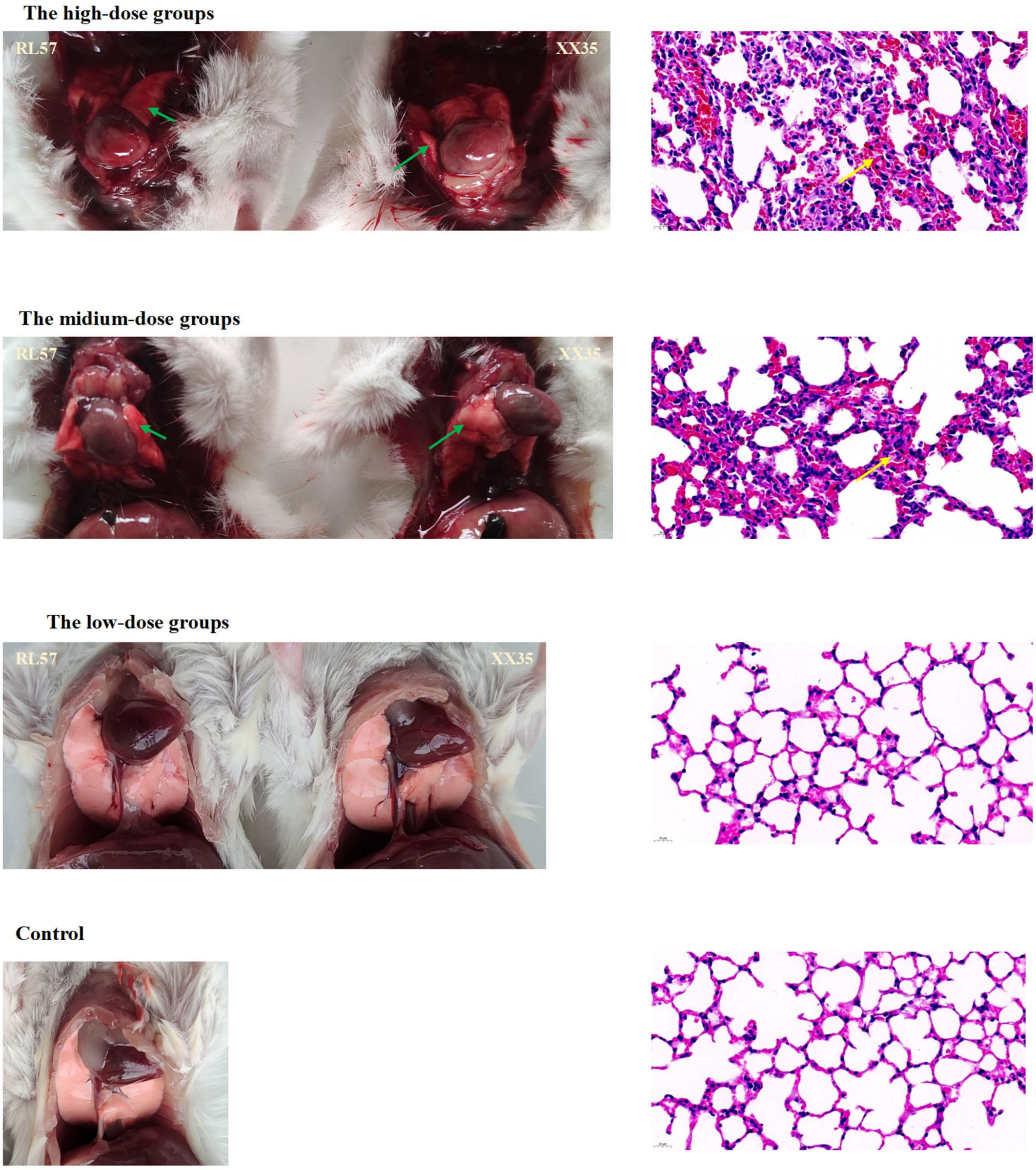

Supplementary Figure 6. Transcriptomic analysis between XX35 and RL57. **(A)** Principal component analysis (PCA) of transcriptomic profiles, demonstrating distinct clustering patterns between strains. **(B)** Transcriptomic volcano plot: Differentially expressed genes (|$\log_2$FC| >1, P <0.05) with RL57-specific upregulation (red) and downregulation (blue). **(C)** Gene Ontology (GO) enrichment analysis highlighting differentially regulated biological processes. (**D**) KEGG pathway enrichment of transcriptomic DEGs (top 8 terms shown).

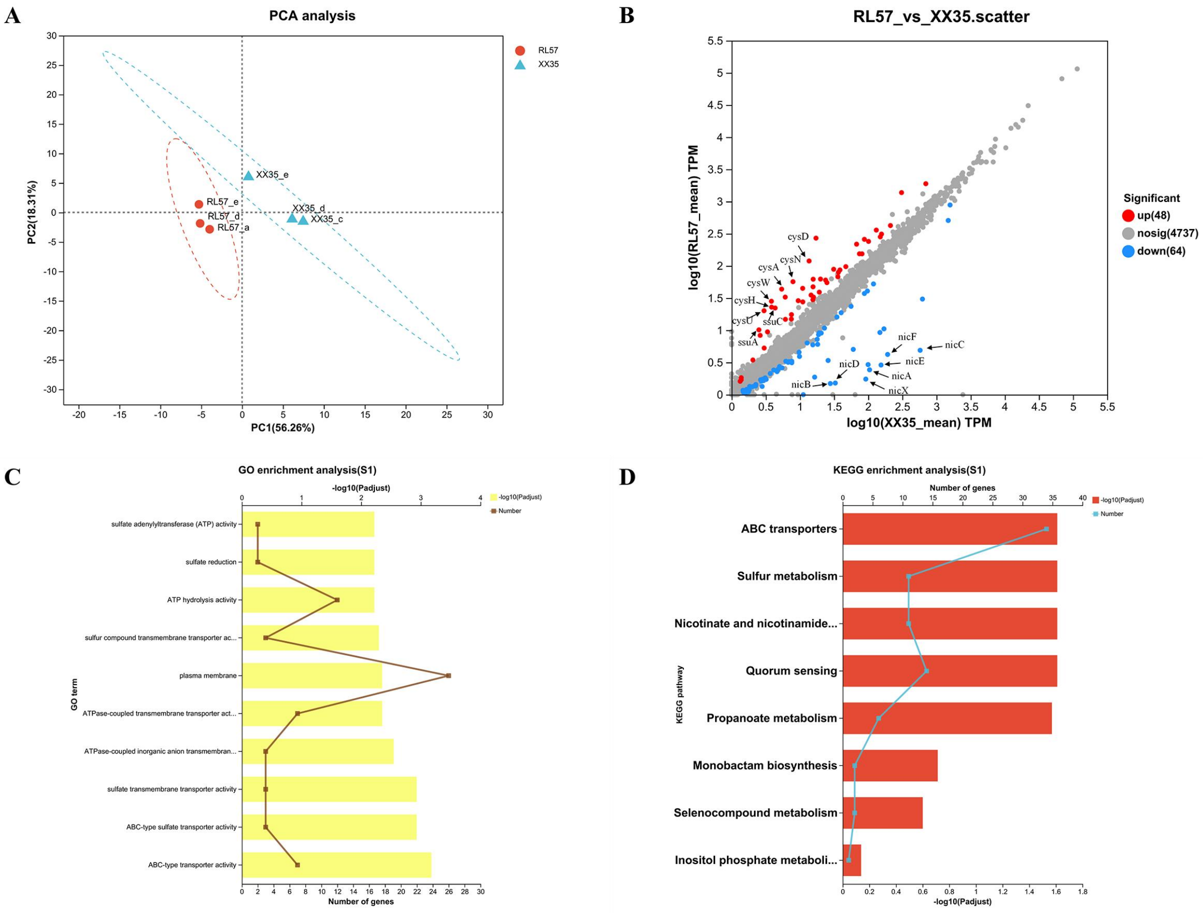

Supplementary Figure 7. Gene expression of XX35 and RL57 by qRT-PCR.

Relative expression levels of genes were determined using the $2^{-\Delta\Delta CT}$ method. Error bars represent the standard deviations of three biological replicates. Statistical analysis was performed using a two-tailed Student's t test. *, P value < 0.05; **, P value < 0.01; ***, P value < 0.001; ****, P value < 0.0001.

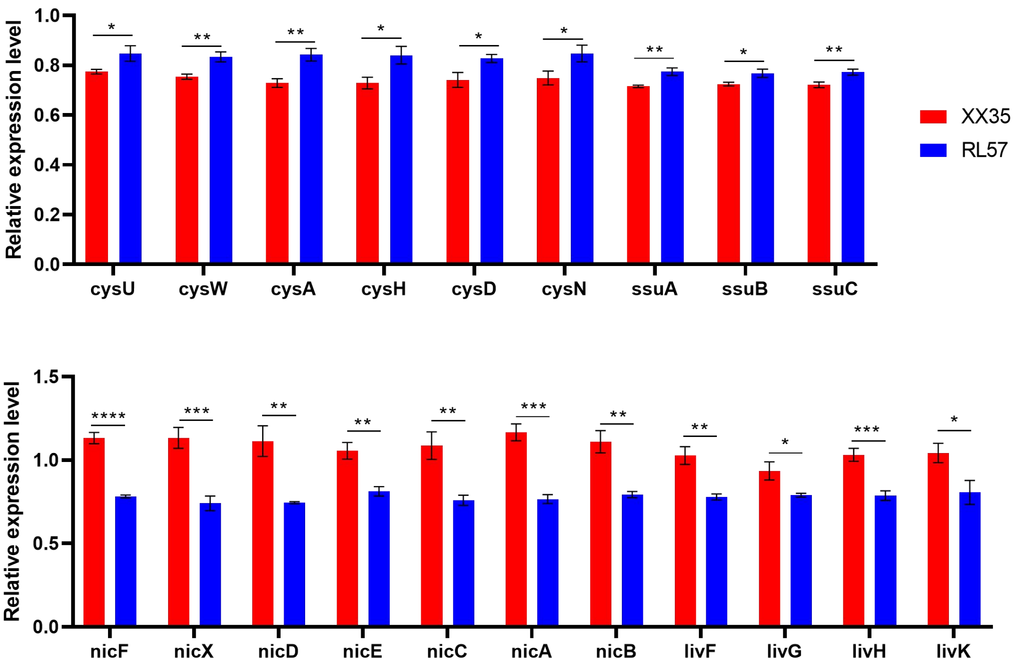

Supplementary Figure 8. Proteomics analysis between XX35 and RL57. (**A**) Proteomic PCA revealing strain-specific protein expression signatures. (**B**) Proteomic volcano plot: Differentially expressed proteins (fold change >1.2 or <0.8, P<0.05) with RL57-specific expression shifts. (**C**) KEGG enrichment analysis of DEPs. Pathway names are displayed along the x-axis. Bar height represents enrichment significance, while the right y-axis indicates gene counts.

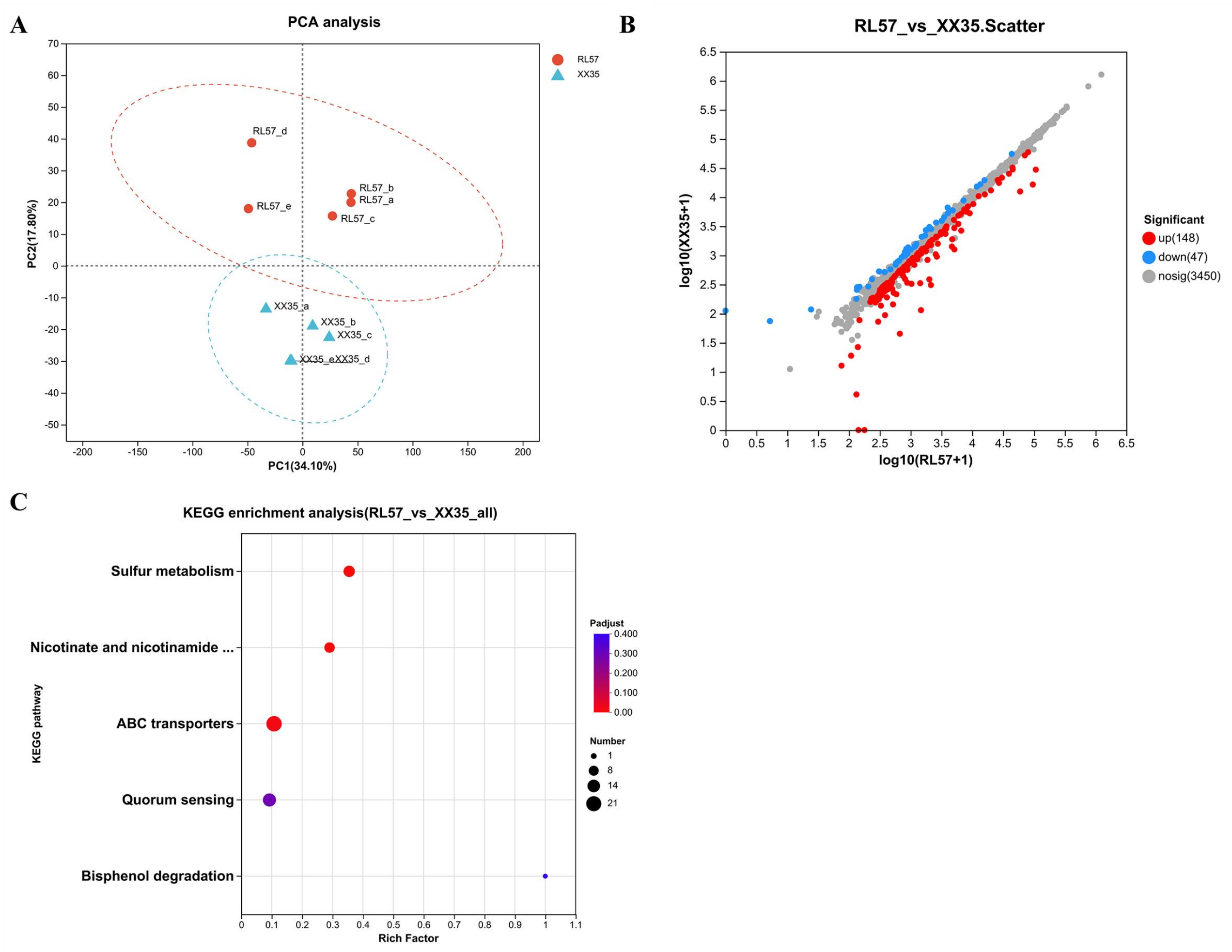

Supplementary Figure 9. PRM-proteomic validation concordance analysis.
The x-axis displays protein accession numbers validated by both PRM and proteomic profiling. The y-axis indicates fold change (FC) values. A dashed red line marks FC = 1. Concordant regulation is defined by bars positioned uniformly above (upregulated) or below (downregulated) the reference line; discordant bars (opposing directions between methods) denote validation inconsistency.

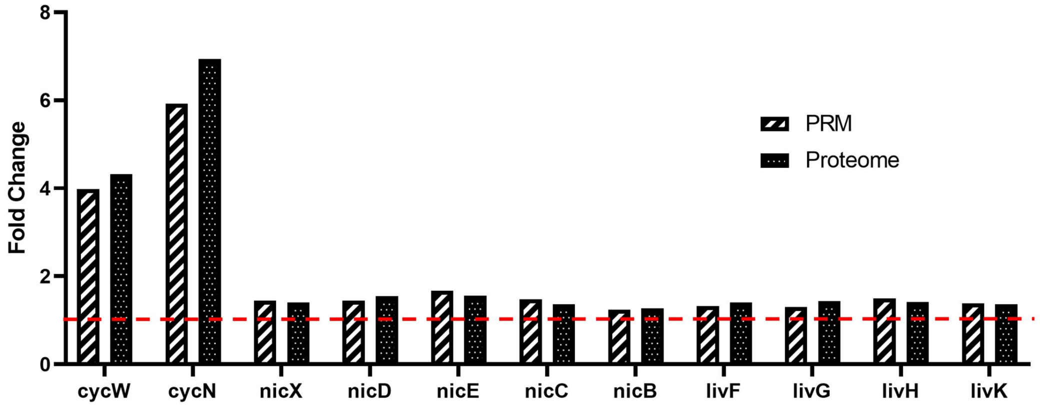

Supplementary Figure 10. Metabolic divergence between porcine isolate XX35 and human-adapted RL57.

(A) Multivariate analysis revealed distinct metabolic clustering through PCA/PLS-DA modeling. **(B)** Volcano plot of differentially expressed metabolites (DEMs) with VIP >1, P < 0.05 (red: RL57-enriched; blue: XX35-enriched). **(C)** The metabolites with the top 15 VIP scores obtained from the PLS-DA models. **(D)** KEGG pathway topology analysis. Enrichment of differential metabolites between human-adapted RL57 and porcine isolate XX35. Dot size denotes pathway impact value; color gradient reflects statistical significance.

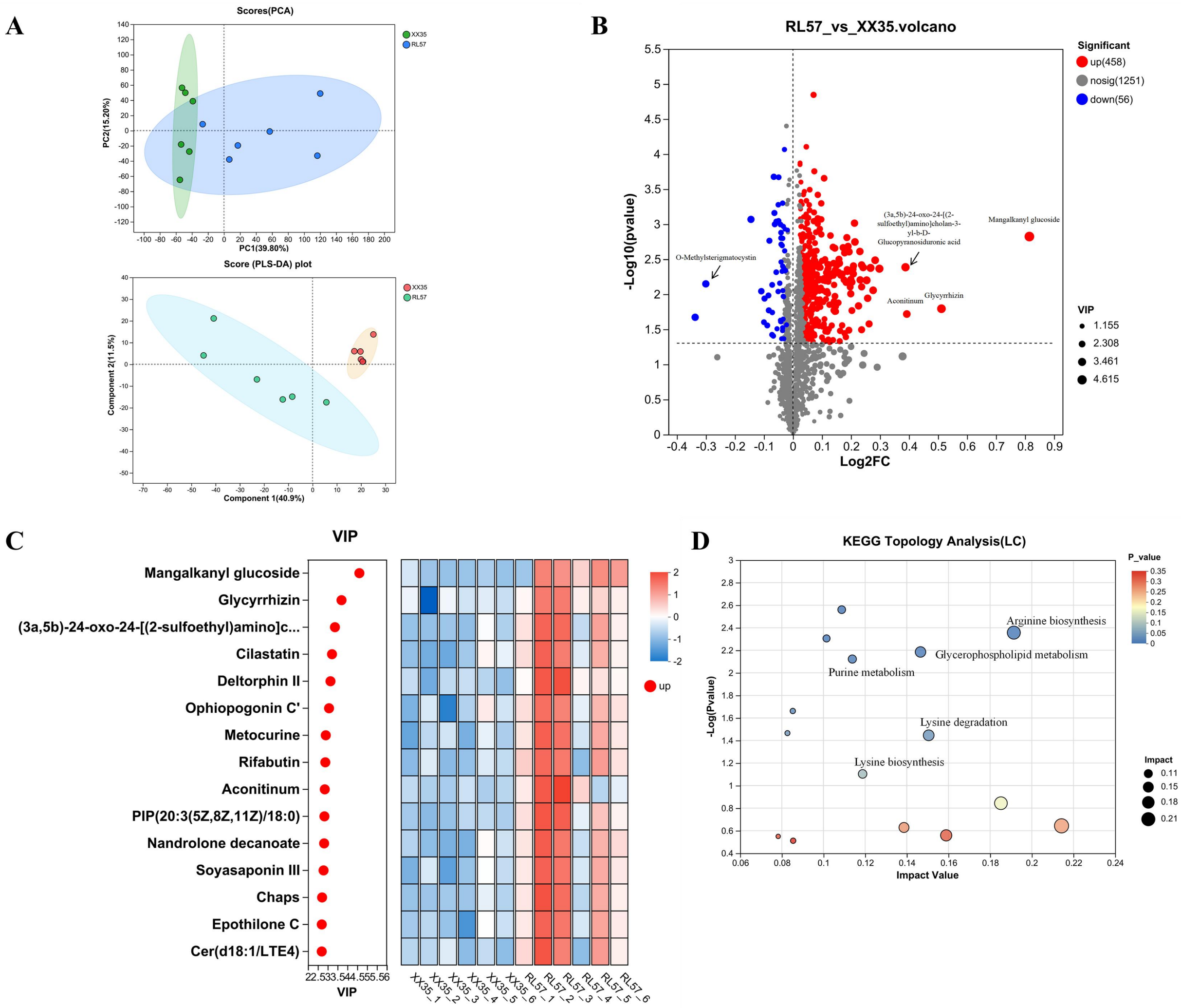

Supplement: SupplementaryDocuments.pdf [file TEMI_A_2637286_SM7349.pdf]
